# Physical activity promotion in Quebec primary schools: equity, intervention practices, and areas for improvement

**DOI:** 10.1093/heapro/daag004

**Published:** 2026-01-30

**Authors:** Erin K O’Loughlin, Maryam Marashi, Robert J Wellman, Annie Pelekanakis, Isabelle Doré, Jennifer L O’Loughlin

**Affiliations:** Centre de Recherche du Centre Hospitalier de L’Université de Montréal (CRCHUM), 850 rue Saint-Denis, Bureau S02-370, Montréal, QC, Canada H2X 0A9; Faculty of Kinesiology and Physical Education, University of Toronto, Toronto, ON, Canada M5S 2W6; Faculty of Kinesiology and Physical Education, University of Toronto, Toronto, ON, Canada M5S 2W6; Department of Population and Quantitative Health Sciences, Division of Preventive and Behavioral Medicine, UMass Chan Medical School, 368 Plantation Street, Worcester, MA 01605, United States; Centre de Recherche du Centre Hospitalier de L’Université de Montréal (CRCHUM), 850 rue Saint-Denis, Bureau S02-370, Montréal, QC, Canada H2X 0A9; Department of Social and Preventive Medicine, Université de Montréal, 7101 avenue du Parc, Montréal, QC, Canada H3N 1X9; Centre de Recherche du Centre Hospitalier de L’Université de Montréal (CRCHUM), 850 rue Saint-Denis, Bureau S02-370, Montréal, QC, Canada H2X 0A9; Department of Social and Preventive Medicine, Université de Montréal, 7101 avenue du Parc, Montréal, QC, Canada H3N 1X9; School of Kinesiology and Physical Activity Sciences, Université de Montréal, 2100, boulevard Édouard-Montpetit, Montréal, QC, Canada H3T 1J4; Centre de Recherche du Centre Hospitalier de L’Université de Montréal (CRCHUM), 850 rue Saint-Denis, Bureau S02-370, Montréal, QC, Canada H2X 0A9; Department of Social and Preventive Medicine, Université de Montréal, 7101 avenue du Parc, Montréal, QC, Canada H3N 1X9

**Keywords:** physical activity, health promotion interventions, school-based, empirically supported components/processes

## Abstract

Schools are pivotal in promoting physical activity (PA) among children through supportive environments and targeted programming. Despite this, in 2024, only 39% of Canadian children met PA guidelines, with inequity linked to socioeconomic status. This study describes the availability of PA activities and facilities in Quebec primary schools according to school deprivation levels and the extent to which school-based PA health promotion interventions (PA-HPIs) incorporated 16 empirically supported components and processes. In Project Prome*SS*, structured telephone interviews were conducted from 2016 to 2019 with key informants (primarily school principals) across 171 Quebec primary schools. PA activities and facilities were generally perceived as available and adequate across all deprivation levels. Nearly all schools (98%) reported PA-HPIs aligned with their mission and values; 96% addressed multiple core competencies; and 86% involved staff in planning. However, only 13% engaged students or peers in intervention development, 35% provided training for internal facilitators, and 35% conducted formal evaluations of PA-HPIs. Availability of PA activities and facilities across deprivation levels may relate to provincial efforts to promote resource equity, although policy impact cannot be inferred from these data. Enhancing underused empirically supported components (student engagement, staff training, program evaluation, and multi-session interventions) could improve the effectiveness and sustainability of school-based PA-HPIs.

Contribution to Health Promotion

- Access to physical activity opportunities is largely equitable across Quebec primary schools, supporting the effectiveness of equity-oriented education and health policies.
- Key implementation gaps—low student engagement, limited staff training, single-session delivery, and minimal evaluation—potentially constrain the impact of physical activity health promotion interventions.
- Results identify evidence-informed strategies to strengthen interventions, including meaningful student involvement, brief theory-based staff training, and structured evaluation.
- This study provides actionable guidance for policymakers, school boards, and practitioners to improve effectiveness, sustainability, and equity in school-based physical activity promotion.

## Introduction

Physical activity (PA) established early in life is a key predictor of lifelong health and well-being. In childhood, regular PA supports healthy development and is associated with improved physical, emotional, and academic outcomes (Centers for Disease Control and Prevention 2024). Healthy PA habits formed early often persist into adulthood, reducing the risk of chronic conditions (Telama 2009). In this context, healthy PA refers to developmentally appropriate, inclusive, and enjoyable movement that promotes physical, psychological, and social well-being and that meets the recommended PA guidelines for youth (World Health Organization 2004).

Despite these benefits, most children fall short of recommended PA levels. The World Health Organization (WHO) recommends that children and adolescents ages 5–17 engage in at least 60 min of moderate-to-vigorous PA daily (World Health Organization 2004). Yet only 39% of Canadian children and adolescents met this guideline in 2024, and just 4% adhered to Canada’s 24 h movement guidelines (ParticipACTION 2025).

Schools are uniquely positioned to help close this gap by offering structured and sustainable PA opportunities. School-based PA healthpromotion interventions (PA-HPIs), educational programs, or activities aimed at increasing PA can effectively reduce sedentary behavior and increase PA participation. PA-HPIs are conceptually distinct from policy, systems, and environmental (PSE) approaches, such as supportive school policies, active transportation infrastructure, and modifications to physical spaces (Schroeder and Kubik 2019). Together, PA-HPIs and PSE strategies can reinforce one another. Unlike optional extracurricular activities, these interventions are typically free of charge, embedded into the school day, and reach all students.

In Quebec, the provincial framework “Au Québec, on bouge!” introduced by the Ministère de l’Éducation, du Loisir et du Sport in 2017, mandates daily PA in schools (Ministère de l’Éducation du Loisir et du Sport 2017). Despite this provincial mandate, schools retain substantial autonomy in choosing which PA-HPIs to implement. As a result, PA-HPIs differ widely in format and scope, leading to considerable variability in implementation practices and overall program quality (Olstad *et al*. 2015). Examples range from classroom activity breaks to school-wide challenges, which have been linked to increased PA and reduced sedentary time (Centers for Disease Control and Prevention 2011; Dobbins *et al*. 2013). Importantly, well-designed PA-HPIs may also help reduce health inequities. In a previous study, we found no significant differences in PA-HPI availability across Quebec primary schools by deprivation level (Riglea *et al*. 2022).

Implementation success also depends on school-level factors, such as leadership, capacity, staff engagement, and school culture (O'Loughlin *et al*. 2022). Barriers such as limited time, training, and resources often constrain feasibility and effectiveness (Weatherson *et al*. 2017). Moreover, the effectiveness of PA-HPIs depends on alignment with empirically supported components and processes of effective interventions (Olstad *et al*. 2015, Hartwig *et al*. 2021, Gosselin *et al*. 2022). Yet little is known about the extent to which school-initiated PA-HPIs reflect these elements.

Although socioeconomic differences in PA environments and policy implementation have been examined, prior applications of evidence-based criteria typically assess researcher-designed or controlled interventions (Kahn *et al*. 2002, Inman *et al*. 2011, Heath *et al*. 2012) rather than day-to-day school-initiated HPIs. Existing studies often evaluate the quality of intervention evaluations rather than the interventions themselves (Fynn *et al*. 2020), and emerging work identifying evidence-based criteria (Brandes *et al*. 2023) has not examined whether these appear in routine school practice.

To address these gaps, we conducted two complementary studies: the first examined whether school deprivation level was associated with (i) availability of PA activities outside school hours, (ii) perceived adequacy of school PA facilities, and (iii) accessibility of community PA resources; the second assessed the extent to which PA-HPIs in Quebec primary schools align with empirically supported components and processes. We first present the shared methodology, followed by study-specific methods and results, and conclude with an overall discussion.

## Methods

Data for this study were drawn from Prome*SS* I, a cross-sectional study conducted between 2016 and 2019 to examine social inequities in school-based HPIs. The sampling frame included 1807 public primary schools in the province of Quebec, Canada. Schools were excluded if they served students with intellectual disabilities or learning challenges, or if they had fewer than 30 students, since these schools are not assigned a government-calculated deprivation index.

Quebec’s public schools are administered by 72 school boards (or service centers), organized by geography and language of instruction (French or English). Three boards were excluded from the sampling frame because they serve Northern and Indigenous communities exclusively, are federally (rather than provincially) funded, and operate under their own governance structures (Gouvernement du Québec Ministère de l’Éducation 2021). Of the remaining 69 boards, 32 (46%) agreed to participate by authorizing recruitment within their jurisdictions. Among 594 eligible schools under the aegis of these participating boards, 291 (49%) were contacted by telephone, and 171 (59% of contacted schools) agreed to participate.

### Data collection

At each participating school, the principal was asked to identify a key informant, either themselves or another staff member, who was knowledgeable about HPIs and had held their position for at least 6 months. Data collection involved structured, two-part telephone interviews conducted by trained interviewers fluent in French and English.

In Part 1 of the interview, data were collected on school characteristics and the availability of HPIs. In Part 2, informants selected a single HPI that had been implemented within the past 3 years for in-depth examination of its planning, implementation, and evaluation processes. Further details on the Prome*SS* I data collection methodology are available elsewhere (Riglea *et al*. 2022).

### Ethical approval

Prome*SS* received ethics approval from the Centre hospitalier de l’Université de Montréal (CHUM) Ethics Review Committee. Written consent for school participation was obtained from school boards, and verbal informed consent was provided by all key informants prior to data collection. The CHUM certificate of ethics approval was submitted to all eligible school boards and to school principals upon request.

### Study 1: access to physical activity opportunities and facilities across socioeconomic contexts in Quebec primary schools

This study assessed whether access to PA opportunities and facilities differed across Quebec primary schools according to their socioeconomic deprivation level, as measured by the Indice de milieu socio-économique (IMSE). Given the pivotal role of schools in supporting children's health and the potential for PA to reduce health inequities, it is critical to understand whether access to PA opportunities varies across school contexts. Although previous research has often assumed that more advantaged schools provide better PA environments (Morin *et al*. 2016), whether this assumption holds in practice remains unclear. This study also considers the extent to which community partnerships may compensate for limited in-school PA resources, particularly in more disadvantaged settings.

#### Study variables

“School deprivation level,” or IMSE, is a composite deprivation score assigned to each public school by the Quebec Ministry of Education. The score assesses the level of deprivation of the school’s location based on parent employment status (both parents employed full-time, one employed full-time, or neither) and maternal education (high school graduate vs. not; Beauchesne 2003). Based on the 2016–17 IMSE, schools were grouped into three categories: highly advantaged (Deciles 1–3), moderately advantaged (Deciles 4–7), and disadvantaged (Deciles 8–10).

“PA opportunities and facilities” are reported by key informants based on six items using the prompt: “Indicate your level of agreement. In your school…” The items were: (i) PA is provided on all days when there is no physical education (PE) class for all students (excluding lunch, recess, or before/after school). Interviewers clarified that this referred to brief, in-class PA sessions (e.g. stretching and jumping jacks); (ii) indoor PA facilities are available to all students outside class time; (iii) indoor facilities meet the needs of all students for PE, extracurricular, and other PA; (iv) outdoor PA facilities are available to all students outside class time; (v) outdoor facilities meet the needs of all students for PE, extracurricular, and other PA; and (vi) students have access to indoor and outdoor PA facilities owned by other schools or community/private organizations (excluding municipal parks). Responses were recorded on a 5-point Likert scale, ranging from strongly agree (1) to strongly disagree (5).

#### Data analysis

To clearly capture agreement, responses were dichotomized as strongly agree/agree vs. all other options (neither agree nor disagree, disagree, and strongly disagree). The proportion of key informants reporting agreement was calculated for each item by school deprivation level.

#### Results

Patterns in key informant responses suggest that moderately advantaged schools most consistently reported favorable conditions for PA opportunities and facilities. These schools showed the highest agreement across several of the six items investigated, particularly for access to indoor and outdoor facilities outside of class time. However, advantages varied by item. For example, highly advantaged schools reported the highest provision of in-class PA on non-PE days (46%) yet reported lower agreement on other items, such as adequacy of outdoor facilities. These patterns suggest that, based on informant perceptions, greater socioeconomic advantage is not automatically associated with more supportive PA environments.

Across deprivation levels, access to indoor PA facilities outside class time was reported by 40% of disadvantaged, 52% of moderately advantaged, and 34% of highly advantaged schools, while access to outdoor facilities outside class time was reported by 77%, 87%, and 80%, respectively. Adequacy of indoor facilities was reported by 80%, 80%, and 74%, and adequacy of outdoor facilities by 71%, 73%, and 57%. Access to PA spaces belonging to other organizations was most common in disadvantaged schools (65% vs. 56% and 62% in moderately and highly advantaged schools), suggesting that community partnerships may help supplement school-based resources (Table 1).

**Table 1:** Key informant perceptions on PA opportunities and access to facilities according to school deprivation level, Prome*SS* I, Quebec, Canada (*n* = 171), 2016–19.

PA opportunities and access to facilities	School deprivation level		
	Disadvantaged % Strongly agree/agree	Moderately advantaged % Strongly agree/agree	Highly advantaged % Strongly agree/agree
PA is provided on all days when there is no PE class to all students (not including activities during lunch, recess, or before/after school)	34	35	46
Indoor school PA facilities are available to all students outside the class timetable	40	52	34
Indoor facilities for PE, extracurricular, and other PA meet the needs of all students	80	80	74
Outdoor school PA facilities are available to all students outside the class timetable	77	87	80
Outdoor facilities for PE, extracurricular, and other PA meet the needs of all students	71	73	57
Access to indoor/outdoor PA facilities belonging to other schools or organizations (excluding municipal parks) is available to all students	65	56	62

### Study 2: alignment of existing school-based physical activity health promotion interventions with empirically supported components and processes

In the second part of the Prome*SS* I interview, key informants selected one HPI for detailed exploration of its components, implementation, and evaluation processes. Of the 171 informants, 99 selected a PA-HPI (Supplementary Table S1 describes these 99 PA-HPIs). We assessed the extent to which these 99 PA-HPIs aligned with empirically supported components and processes related to content, stakeholder engagement, implementation facilitators/barriers, and monitoring and evaluation of PA-HPIs, the efficacy of which had been shown in randomized controlled trials. Alignment was assessed in three steps: (i) synthesizing evidence to identify empirically supported PA-HPI components and processes, (ii) mapping Prome*SS* questionnaire items to these components and processes, and (iii) data analyses.

#### Evidence synthesis

A targeted literature review was conducted to identify recent umbrella and systematic reviews consolidating evidence on components and processes of school-based PA-HPIs that were effective in achieving their objectives (e.g. increasing levels of PA, enjoyment of PA, and PA literacy). The search strategy and review process are described in Table 2.

**Table 2:** Literature review process for identifying empirically supported components and processes in school-based PA-HPIs. Prome*SS* I, Quebec, Canada, 2016–19.

Step	Description
Literature search	PubMed (2010–24) was searched using the following keywords: ((review of reviews) OR (umbrella) OR (scoping)) AND ((physical activity) OR (exercise) OR (movement)) AND (elementary school) AND (intervention) AND (health promotion). A total of 30 articles were identified.
Abstract screening	Abstracts were independently screened by two reviewers (E.K.O., M.M.). Eleven articles were excluded because they focused on weight outcomes, combined PA and nutrition without distinction, or were not relevant to PA in primary school settings. Discrepancies in inclusion decisions were resolved through discussion, with R.J.W. acting as a third-party adjudicator when necessary.
Full-text review	Twelve articles were reviewed in full by both reviewers. Six were excluded due to limited focus on PA, incorrect age group, or setting outside of schools. Six articles were retained (four umbrella reviews, one systematic review, and one review of reviews).
Data extraction	Data were extracted from articles retained (Kriemler *et al*. 2011, Barbosa Filho *et al*. 2016, Craike *et al*. 2018, King *et al*. 2019, Mannocci *et al*. 2020, Alalawi *et al*. 2024) on the following dimensions: (i) PA-HPI components and features (e.g. core competencies addressed, teaching strategies used, multi-session program); (ii) stakeholder engagement (parents/families, teachers/school staff, community groups/school board, students/peers); (iii) implementation enablers and barriers (e.g. compatibility with the school, cost, adaptation to context/demographics); and (iv) monitoring, evaluation, and outcomes (e.g. integration into the school plan, formal evaluation).
Reliability/consensus	Data extraction was conducted independently by the two reviewers. Discrepancies in extracted content were resolved by consensus with input from R.J.W. and J.L.O.

Nine categories of empirically supported components and processes were identified in the literature synthesis: (i) animators (i.e. those tasked with delivering the PA-HPI to students) had the necessary training and competencies; (ii) stakeholder involvement, including parents/families, school staff, community organizations, school boards, and/or students/peers; (iii) intervention incorporated multiple components (e.g. addressed several core competencies and/or aligned with other school initiatives); (iv) sufficient duration and frequency (e.g. a multi-session program rather than a one-off event); (v) use of multiple and innovative teaching strategies (e.g. group discussion, role-play, and peer education); (vi) compatibility with the school’s mission, values, and context; (vii) institutionalization of the intervention (e.g. integration into the school’s official plans); (viii) formal evaluation of the intervention; and (ix) use of theory-based approaches.

#### Mapping Prome*SS* items to empirically supported components/processes

To assess the degree of alignment between existing PA-HPIs and those that had attained their objectives in randomized controlled trials, each component and process identified in the literature review was mapped to corresponding items from the Prome*SS* questionnaire. Since Prome*SS* did not assess whether PA-HPIs were based on behavioral or implementation theories, the theory-based component was excluded from the alignment criteria. This mapping is presented in Table 3.

**Table 3:** Indicators for empirically supported components and processes of PA-HPIs, corresponding questionnaire items from the Prome*SS* questionnaire, and coding of response options for analysis (Prome*SS* I, Quebec, Canada, 2016–19).

Category Components and processes	Description of category/questionnaire item(s)	Coding to indicate endorsement in the analyses
**Animator^a^ training and competencies**	**Indicators assessed whether animators (internal or external) received training and had the required competencies to deliver the intervention**	
1. Training of animators internal to school 2. Training of animators external to school	Prior to implementation, training was provided to animators of (PA-HPI). Animators had the skills required to deliver (PA-HPI)	No (neither agree nor disagree, disagree, strongly disagree), yes (strongly agree, agree)
**Stakeholder involvement**	**Assessed participation of parents/families, school staff, community organizations, school boards, and students/peers**	
3. Parents/families	Were families invited to participate in (PA-HPI)?	No, yes
4. Teachers/school staff	(PA-HPI) animators were…? Check all that apply: (i) homeroom teachers, (ii) other teachers, (iii) students/peers, (iv) school health professionals (e.g. nurse, dental hygienist, etc.), (v) other, (vi) external health professionals (e.g. physician), (vii) members of a community organization, (viii) CEGEP^b^ or university students, (ix) other	No, yes if 1, 2 (teachers) or 4 (school health professionals) was endorsed
5. Community agencies and/or school board	Were community groups invited to participate in [PA-HPI]? Was the school board/service center involved in implementing [PA-HPI]?	No, Yes if either was identified
6. Students/peers	(PA-HPI) animators^a^ were …? Check all that apply: (i) homeroom teachers, (ii) other teachers, (iii) students/peers, (iv) school health professionals (e.g. nurse, dental hygienist, etc.), (v) other, (vi) external health professionals (e.g. physician), (vii) members of a community organization, (viii) CEGEP^b^ or university students, (ix) other	No, Yes if 3 (students/peers) was endorsed
**Multiple components**	**Determined whether the PA-HPI was implemented alongside other school initiatives and addressed multiple core competencies**	
7. Complementary initiatives in the school during PA-HPI implementation	Were there any other initiatives occurring in your school before or around the same time as (PA-HPI) that addressed the same health and well-being issue as (PA-HPI)? Check all that apply: (i) media campaign (e.g. posters, distribution of leaflets, social media, etc.), (ii) assemblies, (iii) extracurricular activities, (iv) linking to services offered by external organization, (v) infrastructure (e.g. installation of bike racks), (vi) social environment (e.g. increased surveillance, support to students, etc.), (vii) school policy (e.g. nutrition, PA, bullying, etc.), (viii) school day care service activities, (ix) special events, (x) other	No, yes if any was endorsed
8. Multiple core competencies addressed by intervention	Were any of the following core competencies incorporated into (PA-HPI)? Check all that apply: (i) self-esteem, (ii) managing emotions and stress, (iii) positive interactions with others, (iv) self-awareness, (v) learning to say “no,” (vi) asking for help, (vii) informed lifestyle choices, (viii) adoption of prosocial choices, (ix) management of prosocial choices, (x) social engagement, (xi) other (specify)	No, yes if more than one was endorsed
**Duration and frequency**	**Evaluated whether the intervention was a sustained, multi-session program rather than a one-time event**	
9. Multi-session program (not single event)	(PA-HPI) was a(n) …? (i) special event (e.g. health fair, guest speaker at an assembly, etc.), (ii) pedagogical activity, (iii) learning and evaluation situation, (iv) program, (v) other (specify)	No, yes if 4 (program) was selected
**Innovative/multiple teaching strategies**	**Assessed use of interactive and social constructivist teaching methods, and whether more than one strategy was employed**	
10. Innovative strategies employed	What type of teaching strategy was used for (PA-HPI)? Check all that apply: (i) lecture strategies: presentations, demonstrations; (ii) individual work: independent practice; (iii) interactive teaching strategies: group discussion, role-play, modeling; (iv) social constructivist teaching strategies: peer education, tutoring, collaborative, and cooperative learning; (v) other (specify)	No, yes if 3 (interactive) or 4 (social constructivist) was endorsed
11. Multiple teaching strategies employed	What type of teaching strategy was used for (PA-HPI)? Check all that apply: (i) lecture strategies: presentations, demonstrations; (ii) individual work: independent practice; (iii) interactive teaching strategies: group discussion, role-play, modeling; (iv) social constructivist teaching strategies: peer education, tutoring, collaborative, and cooperative learning; (v) other (specify)	No, yes if more than one was endorsed
**Compatibility**	**Measured the extent to which compatibility with the school’s mission, values, and context was considered; included tailoring and cost considerations**	
12. Consideration of compatibility in selecting/developing PA-HPI	How important were each of the following in the development of (PA-HPI) in your school? (i) Compatibility with the values and mission of your school, (ii) compatibility with the school context	No (not important), yes (extremely, very highly, highly, important)
13. PA-HPI tailored to school context/demographics	Prior to implementation, did your school make any modifications to (PA-HPI)? (i) No modifications were made (it could be used as is), (ii) no modifications were made (it was already tailored to our school, (iii) no modifications were made (other reason), (iv) yes (minor modifications), (v) yes (major modifications), (vi) yes, but don’t know if they were major or minor modifications, (vii) don’t know (an external agency implemented the intervention in our school); did (PA-HPI) change during its implementation? (viii) did not change at all, (ix) underwent minor modifications, (x) underwent major modifications, (xi) changed completely, (xii) don’t know (an external agency implemented the intervention)	No, yes if any modification/change endorsed
14. Consideration of costs in adopting/implementing PA-HPI	How important was cost in the development of (PA-HPI) in your school?	No (not important), yes (extremely, very highly, highly, important)
**Institutionalization**	**Assessed whether the PA-HPI was written into the school’s formal plans**	
15. PA-HPI institutionalized in school plan	Is (PA-HPI) explicitly written into your school’s orientations (e.g. the educational project, the success plan, or others)?	No, yes
**Evaluating outcomes**	**Assessed whether the PA-HPI was formally evaluated, particularly in terms of outcomes**	
16. PA-HPI formally evaluated	Did your school do any of the following to evaluate (PA-HPI)? (i) Hold regular meetings, (ii) obtain feedback from the (PA-HPI) animators, (iii) document the extent to which implementation was carried out in accordance with the plan, (iv) document the number of students participating in the (PA-HPI), (v) document the barriers and facilitators to implementation, (vi) formally evaluate the outcomes of the (PA-HPI)	No, yes if 6 (formally evaluate) was endorsed
**Theory-based interventions^c^**	**No data were collected on this category in PromeSS**	

^a^Animators were defined as the individual(s) tasked with delivering the PA-HPI.

^b^CEGEP: Collège d’enseignement général et professionnel (a public school providing the first level of post-secondary education, similar to a junior or community college in the US).

^c^Prome*SS* did not inquire about theory-based interventions.

#### Data analysis

Response options for each of the 16 Prome*SS* questionnaire items matched to the indicators for empirically supported components and processes of effective PA-HPIs were dichotomized (e.g. agree/strongly agree vs. all other responses). For some indicators (e.g. use of multiple teaching strategies or core competencies), multiple response options had to be selected to meet the endorsement threshold (e.g. two or more teaching strategies). For each component and process, the proportion of PA-HPIs that met the endorsement criteria was computed.

#### Results

As evident in Table 4, almost all informants endorsed consideration of the compatibility of the PA-HPI with the school and the PA-HPI addressing multiple core competencies. More than four-fifths of informants reported that teachers and school staff animated the PA-HPIs and that complementary initiatives were present in the school during PA-HPI implementation. At least 70% reported inclusion of innovative teaching strategies in the PA-HPI and involvement of community agencies and/or school boards in PA-HPI implementation, while at least 60% reported considering costs, engaging parents or families, and tailoring the PA-HPI to the school. Fewer than half of informants endorsed presentation of the PA-HPI as a multi-session program, using multiple teaching strategies, or institutionalizing the PA-HPI in the school plan. While all seven schools that employed external animators judged them to be adequately trained, only 35% reported training internal animators. A similarly low percentage reported formally evaluating the PA-HPI, and only 13% involved students/peers in implementation.

**Table 4:** Proportion of existing school-based PA-HPIs that attained each empirically supported component or process (PromeSS I, Quebec, Canada, 2016–19).

Empirically supported component/process	*n* (obs)^a,b^	Endorsed indicator^c^ *n* (%)
Animator^d^ training and skills		
1. Training provided to internal animators^e^	86	30 (35)
2. External animators had necessary skills^e^	7	7 (100)
Stakeholder involvement		
3. Parents/families	84	50 (60)
4. Teachers/school staff	92	79 (86)
5. Community groups or school board^f^	99	69 (70)
6. Students/peers	92	12 (13)
Multiple components		
7. Complementary initiatives during PA-HPI	93	78 (84)
8. Multiple core competencies addressed	93	89 (96)
Duration and frequency		
9. Multi-session program	93	39 (42)
Teaching strategies employed		
10. Innovative strategies used	99	74 (75)
11. Multiple strategies used	93	45 (48)
Compatibility with school		
12. Compatibility considered	85	83 (98)
13. Tailored to school context	99	66 (67)
14. Cost considered	85	51 (60)
Institutionalization		
15. Incorporated into school plan/curriculum	91	41 (45)
Evaluation		
16. Outcomes formally evaluated	93	33 (35)

^a^ *n* = 99 PA-HPIs were identified as having PA as a primary focus.

^b^ *n* = number of informants who responded to each item.

^c^Percentages are rounded to the nearest whole number.

^d^Animators are individuals who delivered the PA-HPI. Internal animators included teachers, school professionals, or peers; external animators included health professionals, community organization members, or college/university students. Seventy-three schools used only internal animators, 7 used only external animators, and 13 used both.

^e^“Yes” reflects strong agreement or agreement with the item.

^f^Seventeen schools (21%) involved the school board specifically.

## Discussion

This study examined key informants’ perceptions of PA opportunities within and around Quebec primary schools and the extent to which school-based PA-HPIs incorporate empirically supported components and processes. The findings suggest that while schools report equitable access to PA infrastructure according to school deprivation level, substantial gaps in the use of evidence-supported practices may limit the effectiveness and sustainability of PA-HPIs.

Study 1 assessed access to PA opportunities and facilities across socioeconomic contexts. Informants generally perceived PA-related physical infrastructure as adequate across schools, regardless of deprivation level. However, limited access during evenings, weekends, and holidays emerged as a consistent constraint. No differences were observed across deprivation levels in perceived access to PA opportunities outside school hours, indoor facility adequacy, or availability of nearby community-based PA resources. This is noteworthy given the well-established association between socioeconomic disadvantage and poorer health outcomes (Gautam *et al*. 2023). One possible underpinning is Quebec’s Healthy Schools approach, which promotes context-specific implementation of HPIs across all schools. A 2015 systematic review found that school-based interventions with environmental components were more likely to reduce health disparities, whereas education-only strategies sometimes exacerbated them (Moore *et al*. 2015). Of note, this study captured perceived availability and not actual student use or satisfaction. Future work should incorporate student perspectives to determine whether available resources are genuinely accessible and responsive to their needs.

Study 2 examined the use of empirically supported practices in PA-HPIs. Several strengths were evident: nearly all schools aligned interventions with their mission and values, targeted multiple competencies, and involved staff in planning. However, these perceived strengths should be interpreted cautiously, and notable gaps remain.

Only a small proportion of schools involved students in PA-HPI design or delivery, which could limit relevance, acceptability, and engagement. WHO recommends meaningful youth involvement to ensure that interventions reflect students’ needs (World Health Organization 2021). Although empirical comparisons between youth-led and adult-led PA-HPIs are limited, a scoping review by Christensen *et al*. (2021) indicates that peer-led approaches can improve PA outcomes particularly when grounded in Social Cognitive Theory and when peer leaders possess strong communication and leadership skills. Future research should explore feasible models for sustained student involvement.

Another important finding was that fewer than half of schools provided training for internal facilitators (e.g. teachers). This suggests a potential skills and capacity gap that could undermine intervention fidelity and sustainability. Without adequate training, schools may rely on external partners, whose availability may fluctuate (Herlitz *et al*. 2020). Effective training programs should be short, theory-based, and include active components such as modeling, practice, feedback, and clear behavioral goals (Ryan *et al*. 2022). Strengthening internal capacity is essential for safe, effective, and sustainable PA-HPI delivery.

A third area for improvement concerns the structure and duration of PA-HPIs. Only 42% of schools implemented multi-session interventions; most relied on single-session or one-off events. This is concerning given strong evidence that sustained, multi-session interventions outperform one-time activities in promoting PA among children (Dobbins *et al*. 2013). Multi-session interventions foster routine, skills, and continuity and are more easily institutionalized within school systems (Herlitz *et al*. 2020). In contrast, single events may generate enthusiasm but lack sufficient dosage or reinforcement for lasting behavior change. Encouraging schools to adopt multi-session models (even modest ones) could substantially improve impact and sustainability.

The low frequency of formal evaluation reported by schools is another challenge. Without systematic evaluation of feasibility, fidelity, or outcomes, schools may struggle to improve programming, make evidence-based decisions, or justify investment (Cassar *et al*. 2019). Structured evaluation frameworks, such as the CDC’s School Health Index (Centers for Disease Control and Prevention 2017), can support school-level monitoring and refinement. Incorporating basic evaluation training into professional development may reduce reliance on external researchers and build long-term internal capacity.

### Implications for policy, practice, and research

These findings offer several actionable insights. The perceived equity in access to PA facilities suggests that Quebec’s emphasis on resource equity may be meeting its goals. However, PA opportunities during noninstructional hours remain limited, indicating a need for stronger collaboration between school boards and community partners to expand access to facilities outside school time.

Improving PA-HPIs requires greater adoption of evidence-supported practices. Short, theory-based staff training can help build internal capacity and reduce reliance on external partners. Meaningful student involvement should be encouraged to enhance relevance and uptake. Given the strong evidence base, policies should prioritize multi-session interventions over single events. Future research should examine barriers to adopting sustained PA-HPIs and identify strategies that support their institutionalization across diverse settings. Finally, schools should be encouraged to implement structured evaluation tools to support program refinement, accountability, and long-term sustainability.

### Strengths and limitations

This study has several strengths. It draws on a large, provincially representative sample of Quebec primary schools and examines both PA opportunity availability and alignment with evidence-supported implementation practices. Quebec’s unique policy environment (mandatory daily PA with local autonomy) offers a natural context to test whether perceived PA environments and intervention practices vary by deprivation level. Use of knowledgeable key informants yielded detailed, context-specific insights. Indicators of evidence-supported intervention design were derived from up-to-date umbrella and systematic reviews, ensuring strong scientific grounding. Although not theoretically novel, translating contemporary evidence-based criteria into a practical checklist provides a useful decision tool for schools and school service centers operating under real-world constraints.

Limitations include the modest response rate, although participating schools resembled all Quebec primary schools (Riglea *et al*. 2022). Reliance on a single informant per school may not fully capture the diversity of perspectives, although informants received materials in advance and could consult others. Assessing only one PA-HPI per school limits understanding of the full range of activities. Lack of student input prevents assessment of how perceived availability translates into actual engagement. Data were collected between 2016 and 2019 and may not reflect changes following the COVID-19 pandemic; however, many PA-HPIs identified remain active province-wide. Prome*SS* collected updated data in 2025, enabling future research to examine changes over time.

## Conclusion

PA facilities and activities are generally perceived as equitably available across Quebec primary schools regardless of socioeconomic deprivation, potentially reflecting effective provincial equity policies. However, limited PA opportunities outside PE days and restricted access to indoor facilities during noninstructional hours persist.

Several empirically supported components, and especially student engagement, staff training, multi-session structures, and formal evaluation were inconsistently applied. Addressing these gaps could substantially enhance the effectiveness, sustainability, and equity of school-based PA promotion. Future research should examine strategies to build internal staff capacity, meaningfully involve students, institutionalize evaluation processes, and embed multi-session interventions into school routines to ensure that PA-HPIs achieve their intended health impacts.

## Supplementary Material

daag004_Supplementary_Data

## Data Availability

Data are available upon request.
