# Supplementary material for: Physical activity promotion in Quebec primary schools: equity, intervention practices, and areas for improvement
Source: Health Promot Int. 2026 Jan 30;41(1):daag004. doi: 10.1093/heapro/daag004 (PMC12857214; doi:10.1093/heapro/daag004)
Supplement: daag004_Supplementary_Data [file daag004_supplementary_data.docx]

**Supplementary Material**

**Table S1. Key informants’ descriptions of 99 PA-HPIs implemented in Prome*SS* schools in the past year. Prome*SS* I 2016–2019**

|  | PA-HPI | Description |
| --- | --- | --- |
| 1 | Le défi des Cubes (n=16) | The Energy Cubes Program encourages Quebec primary schools to adopt healthy lifestyle habits by promoting physical activity. For every 15 minutes of physical activity, students earn one Energy Cube. Schools can freely sign up, and the designated coordinator informs teachers, students, and parents about the challenge, encouraging ongoing participation.  A class leader—who can be a teacher, parent, or student—is responsible for tracking each student’s Energy Cubes in a logbook and submitting weekly totals to the school’s coordinator. Participating schools receive a printed Student Logbook free of charge (while supplies last), or they can download a condensed version from the website.  Families can also participate by adding cubes to their children’s prize tally. Schools earn entries into a prize draw based on the average number of cubes per student.  Prizes include:   - *La Grande récompense*: A celebration where 4,200 winning students spend a night at Montreal’s Olympic Stadium and a day at La Ronde amusement park. - *The Véhicube*: A touring interactive program visiting elementary schools to teach students about healthy eating and physical activities.   <https://cubesenergie.com/> |
| 2 | BOKS (n=3) | Non-profit organization created by Reebok. Free program for primary school children. It is designed to help children start their day with physical activity to stimulate them both physically and psychologically, giving them the energy boost they need for a productive day of learning. BOKS is based on the principles of Dr John Ratey's book “Spark”, which suggests that “exercise is the most useful tool we have to keep our brains working properly”. With financial support from a multi-sectoral partnership with the Public Health Agency of Canada, BOKS reinforces PHAC's commitment to helping all children meet the doctor-recommended 60 minutes of daily moderate to vigorous physical activity. Children typically meet 2-3 mornings a week, for 45-minute training sessions. These cover different themes and functional physical abilities, incorporating everyday movements and games.   - Gathering and warm-up: Once attendance has been taken, the coach gives an overview of the day's lesson plan and the week's skills. - Running activities: The coach leads a fun activity that requires the child to run, as running is an integral part of all classes. - Skill of the week: Each week, the child must practice developing a skill, such as pull-ups, sit-ups or squats. These exercises are integrated into fun relay races or obstacle courses. - Final game: Trainers encourage solidarity and teamwork with an entertaining game. - Recovery and tips: Under the supervision of their coach, children stretch, recover and share the week's nutritional tip (BOKS tip).   Funding :  BOKS is a free physical activity program created by the non-profit Reebok Canada Fitness Foundation for primary school children. It aims to energize kids both physically and mentally to support a productive school day. Inspired by Dr. John Ratey’s book *Spark*, which emphasizes exercise as a key tool for brain health, BOKS encourages children to engage in 45-minute sessions 2-3 mornings per week, focusing on fun and functional physical activities.  Each session typically includes:   - Gathering and warm-up: Attendance, lesson overview, and weekly skill focus. - Running activities: Fun running games, integral to all classes. - Skill of the week: Practice of specific exercises (e.g., pull-ups, sit-ups, squats) incorporated into relays or obstacle courses. - Final game: Team-building and solidarity through entertaining games. - Recovery and tips: Stretching and sharing a weekly nutrition tip.   Funding & Support: BOKS is funded through partnerships with the Public Health Agency of Canada, the Canadian Football League, and the PROPEL Centre for Population Health Effects. The program is offered free, including training and start-up materials, although schools may incur minor costs for supplies like first-aid kits or basic equipment.  BOKS uses two evaluation approaches:   - Process evaluation: Focuses on program implementation via school visits, focus groups, and interviews with coaches, principals, and staff. - Impact evaluation: Measures participants’ physical activity using pedometers/accelerometers, race data, surveys, attendance, and behavioral logs.   <http://bokskids.ca/fr/> |
| 3 | On bouge au cube (n=2) | On days without physical education classes, teachers provide an additional 30 minutes of physical activity once daily. Other fun activities are organized during this time to help students accumulate at least 60 minutes of physical activity each day. <https://cubesenergie.com/fr/ecole-primaire-du-quebec/defi/idees-pour-bouger>  <https://centdegres.ca/ressources/a-l-ecole-on-bouge-au-cube-une-infographie-pour-comprendre-la-mesure-15023> |
| 4 | On bouge! | This program encourages students to log their physical activities for one month, with regular follow-up by the teacher. The program concludes with a reward activity—field hockey. |
| 5 | Vélo-Tour | A cycling course with routes tailored to different levels and difficulties involves the entire school and parent volunteers. During lunch, participants enjoy a healthy picnic snack. The activity is supported by the local community bike organization. |
| 6 | Vélo-thon | The school participates in the Boucle Pierre Lavoie, a 130 km bike ride. To engage the entire school community, stationary bikes are set up in the schoolyard during June. Throughout the day, each class collectively pedals the 130 km distance, with each student cycling about 1–2 km. Teachers, parents, and community members from local groups and businesses are also invited to join, with the event serving as a fundraiser as well. |
| 7 | Marche du Défi Pierre-Lavoie | The school has registered for the Pierre-Lavoie Challenge and provided each student with a participation booklet. To support students in reaching their challenge goals, the school organizes a weekly 20-minute group walk around the neighborhood every morning, with the entire school participating together. |
| 8 | Tournois activité physique | Offered to grades 3 through 6 in collaboration with four nearby schools, this program hosts intramural games (cosom field hockey, soccer, basketball) every 10 days at 5 p.m., followed by competitive tournaments. Students receive healthy snacks and participate in workshops on nutritious snacks and sports drinks. |
| 9 | Terry Fox Run | For over 30 years, the Terry Fox Foundation has been dedicated to realizing Terry’s vision of a cancer-free world. The Terry Fox Run invites participation from people of all ages, physical abilities, and socioeconomic backgrounds. A program tailored for primary and secondary schools across Canada encourages students to raise funds through online donations, pledge sheets, or onsite contributions on race day. Teachers are encouraged to share Terry’s story to raise awareness, and students can train outside class hours for the final race. <https://terryfox.org/terry-fox-run/> |
| 10 | Système D | This program encourages students from different schools to ride bikes together with staff outside school hours, fostering social connection. Inspired by Pierre Lavoie’s 1000 Kilometers initiative, the program includes two dedicated days focused on healthy lifestyle habits and physical effort. |
| 11 | Semaine de l’éducation physique | This activity is part of Physical Education Month and aims to promote healthy lifestyle habits. It is managed by tenured physical education teachers as an annualized component of their duties. |
| 12 | Récréations Animées | This activity involves TES students and 5th-6th graders leading games and exercises for other students during recess, mornings, or after school every day in May and June. Additionally, parents occasionally organize evening activities. |
| 13 | Projet Éduc | The program aims to introduce students at all levels to various sports, offering 2 to 4 activities per year. Each activity is accompanied by a session on healthy snacks that complement physical exercise. The sports by grade are as follows:   - Kindergarten: introduction to skiing - Grade 1: skating and introduction to skiing - Grade 2: skating and swimming - Grade 3: cycling with parents and futsal - Cycle 3: snowboarding, golf, badminton, and field hockey |
| 14 | Planificateur Familial | A magnetic calendar is given to all students to promote school motivation, healthy nutrition, physical activity, and overall wellness. Monthly health promotion messages and calendar reminders are broadcast over the school intercom. |
| 15 | Nutritional Police | During lunch, a teacher observes students’ meals and selects those with less balanced lunches to provide an educational session on healthy eating and physical activity. |
| 16 | Marchethon et préparation d’une salade de fruits | A relatively long walk, usually to raise funds for a particular cause |
| 17 | Marathon de l’école | One afternoon in May, the entire school participates in a walk/run circuit. Along the route, stations staffed by a nurse offer water, fruits, and vegetables to participants. Parents are also invited to join the event. |
| 18 | Les journées de compétitions sportive (n=2) | Developed by a physical education teacher within a regional school board, this program focuses on physical activity, healthy eating, personal hygiene, and safety. It is offered twice a month throughout the year to grades 3 through 6 and is also available at three other schools within the same school board that share the same principal. |
| 19 | La traversée du Canada en vélo stationnaire/ projet vélo Canada | Students work together to pedal a total of 6,000 km by the end of the school year, taking voluntary 20-minute turns on a stationary bike. This initiative encourages healthy lifestyle habits, especially physical activity, which in turn improves concentration and academic performance. The classroom is adapted to include a desk and/or stationary bike, and progress is tracked on a large map showing a virtual journey across Canada, helping reinforce geography skills. Along the way, students participate in culinary workshops to learn about different foods and their nutritional benefits. |
| 20 | Jouer et vivre en harmonie | Developed by specialized educators and inspired by an existing intervention, this program is funded by Fondation Jasmin Roy in partnership with Institut Pacifique. It promotes healthy lifestyle habits and encourages play as a means to foster pro-social behaviors. |
| 21 | In School, We Move | This pilot project, supported by the school board and Ministry initiatives, aims to ensure children are active for at least 60 minutes daily. To increase physical activity during class time, the school uses smartboard videos that guide students through movements. Additionally, some classrooms have stationary bikes or pedal attachments on desks. These activities are offered across all grades and classes. |
| 22 | Force 4 (n=2) | The goal is to provide physical activity opportunities beyond physical education classes. The intervention includes in-class video sessions that encourage movement during lessons, the creation of active corridors throughout the school, and numerous outdoor school activities. Financial support is provided by the Ministry. <https://www.force4.tv/> |
| 23 | École en forme et en santé | This program, subsidized by MELS, targets students in Cycle 3 of elementary school. Schools implement projects aimed at teaching healthy lifestyle habits, including regular physical activity and nutritious eating. |
| 24 | Duathlon | Offered to all K-6 students, staff, and parents, this program includes a 6-week training log. Training sessions occur three times a week during school hours and daycare. The program culminates in a community Duathlon Day. |
| 25 | Défi du bois joli | An annual neighborhood run involving the entire school community—students, teachers, and parents. Preparation takes place during physical education classes and daycare. Parents are encouraged to run alongside the children or volunteer. The city and police department assist with supervision and safety. |
| 26 | Défi 100 milles | Launched in 2013, the “100 Mile Challenge” encourages people in Abitibi-Témiscamingue to stay active. Over 5,500 participants have taken part since its inception, engaging in activities such as running, walking, cross-country skiing, snowshoeing, and swimming across Quebec and beyond. Participants aim to complete at least one mile a day for 100 days, often in challenging weather.  Adults pay a $20 registration fee, while youth under 18 and school groups participate for free. The half-challenge “D50M,” offered in January, costs $12.  Participants can personalize their experience by choosing the “Audacious” or “Energetic” categories, which allow additional activities such as elliptical training, rowing, and cycling.  A flexible leave policy permits up to two absences for minor reasons, with a “Force Majeure” leave for serious illness or injury. This honor-based system requires participants to make up missed miles at the end, ensuring everyone has the chance to complete the challenge. |
| 27 | Course DesChênes Toi! | The school board organizes an annual race, originally started by two school principals. It began with a few hundred participants and has grown to attract around 12,000 runners. The entire Sentier school participates, proudly wearing school T-shirts. The event features entertainment, with parents and teachers volunteering or running alongside the children. Students can also join an after-school running club to prepare for the race. <https://www.deschenestoi.com> |
| 28 | Club de course | Developed by enthusiastic teacher-runners responding to student interest, the school running club trains twice a week. About 80% of students formally join, committing to additional weekly training at home. Most sessions occur in the gym, with total laps run tracked and displayed school-wide. The goal is to surpass the previous year’s total laps—last year, the school celebrated reaching 1,000,000 laps. In June, over half the students participate in the fee-based Course Bouge pour ta santé organized by the CISSS. |
| 29 | Calendrier Défi Pierre-Lavoie | The school participates in the Pierre-Lavoie Challenge and organizes a calendar of daily physical activities throughout May. All grades, including special needs and students with disabilities, take part. Activities include gumboot games, extra recess, morning walks, track and field, baseball, relay races, and more. |
| 30 | Ateliers de Parents | Starting in October, every Friday afternoon is dedicated to a variety of school-wide workshops led by parents. Activities rotate but always include one focused on nutrition and one on physical activity, alongside cultural activities. Parents lead sessions based on their interests and expertise, with teachers providing support and organization. This program has been running for over 10 years. |
| 31 | Atelier de sensibilisation à l’alimentation | A weekly one-hour classroom workshop on healthy eating is delivered by teachers according to the school timetable. The program includes lessons on Canada’s Food Guide and hands-on cooking workshops. |
| 32 | Alimentation Saine - Smoothie | The school receives a gift card sponsorship from a local supermarket. Teachers and students in grades 3 and 4 collaborate to design a healthy meal for the entire school to prepare together—this year, smoothies. With guidance from a school board nutritionist, they prepare the meals and deliver a healthy eating presentation to the school. Students also learn about smart shopping, including food pricing and grocery budgeting. Grade 3 students mentor Grades 1 and 2, while Grade 4 students support Grades 5 and 6. |
| 33 | Activité sportive | Created in 2008, this annual High-Performance Initiative (HPI) is a physical activity event held at the end of the school year. Students train for 1.5 months beforehand with their teachers. The program includes a healthy eating component, where physical education teachers discuss nutrition and its relation to training. For the first six years, the event was a triathlon; in the past three years, the format has changed annually. This year’s event is an obstacle course. |
| 34 | Activité physique vs. estime de soi | This intervention targets students with behavioral difficulties and low self-esteem. It involves weekly meetings with the physical education teacher focused on sports activities and awareness-building. |
| 35 | Activité des Loups | This program is based on a psychomotricity model for kindergarten and a high school sports model for Cycle 3. It was developed by the school's principal and physical education teacher, both of whom have experience in psychomotricity. |
| 36 | Course St-Martin | Race organized by several CS schools. |
| 37 | À pied, à vélo, ville active | À pied, à vélo, ville active is a program launched by Vélo Québec in 2005 to promote active transportation. It partners with municipalities and schools across Quebec to encourage children and their parents to walk or cycle to school. The program has two main goals:   1. To shift travel habits on the home–school–work route toward active transportation. 2. To create safer, more supportive environments for walking and cycling—especially for elementary-aged children.   <https://www.velo.qc.ca/programmes-et-campagnes/a-pied-a-velo-ville-active/> |
| 38 | Acti-Récré | Acti-Récré is a recess-based initiative offered since 2003 to all elementary students in nine schools within the school board. Its goal is to foster playground harmony through structured daily activities over several weeks of the school year. The program also promotes the development of students’ interpersonal and leadership skills.  A *Spiritual Life and Community Engagement Animator* (AVSEC), provides training to school staff—including teachers, professional personnel, and daycare workers—and meets weekly with student leaders to offer guidance. The program's volunteer component is recognized by the *Réseau jeunes bénévoles en action*, which honors students who complete 40 hours of service with a certificate at a ceremony each May.  <https://fondationscolairedelaval.com/portfolio/acti-recre-ecole-la-source/> |
| 39 | Attention, enfants en mouvement | Developed in collaboration with the CSSS and daycare technicians, this program was initially offered to kindergarten and Grade 1 students. It promotes a new vision of physical activity and child development by encouraging situations that allow children to engage in forms of play often considered “risky”—such as playing in the rain, climbing, and wrestling. The goal is to support physical development and autonomy through active, exploratory play.<https://enfantsenmouvement.com/> |
| 40 | Fort Plein Air | Originally used in other schools, this program was adapted by the physical education teacher specifically for Grade 6 students. It was funded for the first three years by the Commission scolaire de Laval. The program features interactive strategies with a mix of recurring activities and the introduction of new sports. Activities are led by Grade 6 teachers and supported by external facilitators when needed. |
| 41 | Ma cour, un monde de plaisir | Offered by Jeunesse en Forme (Quebec en Forme), this program encourages widespread student participation in physical activities adapted to their interests and developmental stage. Games are structured with clear time and space guidelines and are designed to maximize physical space use. Students, under adult supervision, help choose and lead activities, manage equipment, and resolve conflicts. Adult supervisors actively support and animate the program.  Student leaders from Grades 3 to 6 serve as playground facilitators, mediators, and first-aiders. The initiative includes resources such as a brochure and external support (e.g., a nurse facilitator). The *Ma cour : un monde de plaisir!* guide, developed by Kino-Québec, supports schools in creating more engaging and well-organized schoolyards by offering planning tools and strategies for implementation.  <https://publications.msss.gouv.qc.ca/msss/fichiers/2017/17-289-02W.pdf> |
| 42 | Patin | Skating outings with a physical education teacher who teaches the students. |
| 43 | Acti-Récréation | An active school recess initiative featuring an Ultimate Frisbee tournament for 4th and 5th grade students. Organized in partnership with Sports Laval and a professional Ultimate Frisbee player, students were taught the rules and participated in a school-based tournament. Eliminated teams and other students were encouraged to attend matches and cheer on their peers, promoting school spirit and engagement. |
| 44 | Mois de l’éducation physique (activité physique) | The school adopted the *Cubes Énergie* program to promote physical activity among students. To help students accumulate more “cubes,” the school implemented several complementary activities, including a “Bingo d’activité” challenge, where an additional physical education class is held and prizes are awarded to two classes that complete the most diverse activities. Other initiatives include a weekly morning aerobics class throughout May and a family sports evening, where parents are invited to participate alongside their children. |
| 45 | Active Leaders | This initiative offers twice-weekly activities that promote elementary students' roles as both participants and facilitators in sports activities. |
| 46 | Structured Recess | Structured activities supervised by the daycare service during recess. Purchase of sports supplies and equipment. |
| 47 | Positive Behavior Program | This school-wide program, for kindergarten through 6th grade, addresses different themes each month to promote positive behavior and counter bullying. Emphasizing positive reinforcement over punishment, students are divided into eight color-coded teams based on grade levels to foster collaboration and cooperation. Teams earn tokens for good behavior, with prizes awarded monthly to the top three teams (e.g., extra activity time or hot chocolate). The program is adapted from a previous intervention that was challenging for teachers to implement. |
| 48 | Alternative Recess | When outdoor recess isn’t suitable for some children, teachers organize and supervise physical activities in the gym to provide a safe space and help prevent bullying. |
| 49 | Projet Sport | Developed by a PE teacher and the principal, this annual project is offered to all students. Every 10 days, on three afternoons, the student body is divided into thirds: each group participates in a one-hour physical activity session during school hours. |
| 50 | Festi-Neige | This program offers children the chance to participate in half-day snow sports three times a year. Cycle 3 students, supported by teachers, lead the workshops and activities. All students from kindergarten to grade 6 take part. Healthy snacks are occasionally provided before some activities. |
| 51 | Anti-Bullying | During Respect Week (Anti-Bullying Week), the school organizes a day of presentations by guest speakers for all students. Everyone also wears pink shirts to show support, as pink is the symbol of anti-bullying. |
| 52 | Winter Boot Camp - Classes blanches | Offered to Cycle 3 students at a Montreal elementary school , this program includes “White Class” days organized by an outdoor center specializing in vacation and leadership activities. Students take part in various outdoor activities designed to develop leadership skills. The school covers the fee of $19 per day, per student. <http://boutentrain.com/classes-natures/classes-blanches/> |
| 53 | Lunchtime Physical Activity | Developed by two physical education teachers from an English Montreal School Board elementary school , this program offers weekly lunchtime gym sessions to all students on a rotating grade basis. Students practice and expand on skills introduced during physical education classes through various activities such as soccer, badminton, and basketball. The sessions are led by the gym teachers. |
| 54 | Triathlon | Developed about 10 years ago by a physical education teacher in collaboration with another school within the same school board, this program now involves all schools in the board. It offers weekly training sessions for the entire school, with sports changing during the winter season. Additionally, the schools organize regular tournaments for various sports throughout the year, culminating in an annual meeting where all participants gather. |
| 55 | École de sport | Developed in 2000 by a school, this program offers indoor and outdoor physical activities during five Friday afternoons in winter. It has since been adopted by several other schools within the school board. |
| 56 | Projet Patin | For the month of January, regular physical education classes are paused and replaced with skating lessons at the local arena. These lessons are led by physical education teachers and parent volunteers, and are available to all students in kindergarten through grade 2. |
| 57 | Acti-Midi | Developed by a physical education teacher, this program involves students in grades 4 to 6 volunteering to lead sports activities for all school levels during lunch. Equipment is also loaned out at recess, with students managing its use. The activity runs throughout the entire school year. |
| 58 | Course École | Offered to students in grades 1 through 6, this program involves year-round training sessions in preparation. Everyone participates — students, teachers, and parents alike. |
| 59 | Projet d’éducation physique | The physical education teacher takes half of the students out for an hour of physical activity daily—9 days out of 10 for younger students (Cycle 1) and 8 days out of 10 for older students (Cycles 2 and 3). The remaining students stay in class for more personalized instruction. Activities include team sports, introduction to new sports, and motor skills workshops. |
| 60 | Jeux Olympiques Spéciaux | Offered to all special education students, this annual event organizes Olympic-style games featuring Paralympic athletes. This year’s focus was swimming. Medals are awarded to winners, celebrating participation and achievement. |
| 61 | Bouger pour réussir | Developed with funding from an anonymous donation, this activity encourages all students to be physically active every morning. Created by the physical education specialist and remedial teacher, its goal is to increase daily movement and improve student engagement and retention. <https://bougerpourreussir.com> |
| 62 | Promotion de l’activité physique | Activities are integrated into class time to encourage all students to be active. Additionally, every Thursday, the physical education teacher leads organized physical activities in the schoolyard during recess. |
| 63 | Départs Actifs | Every school day, year-round, the schoolyard is open and lively for 10 minutes before classes begin. Students participate in various sports activities accompanied by music, all supervised by the physical education teacher. |
| 64 | Club de Course | The school organizes a running club for students in grades 4 to 6 to prepare for a major regional race at the end of the school year. The club meets weekly starting in early spring for group runs. |
| 65 | Défi St-Barth | Every May, students participate in a 30-minute walk/run event at the school’s running track, aiming to cover as many laps as possible. After each completed lap, students receive a wristband as a marker. The school tracks the total laps completed by all participants. To prepare for the event, students practice during physical education classes, lunch breaks, or class time, often including activities like 1 km walks. |
| 66 | On bouge à l’école | Each morning, all students participate in a 15-minute walk, either outdoors when weather permits or indoors during colder conditions. To accommodate this, the morning class time is shortened by 15 minutes. Throughout the year, additional sporting activities are organized, including events like a long-distance bike ride. <https://www.cssdeschenes.gouv.qc.ca/nouvelles/01061> |
| 67 | Health Break | Students have access to designated areas during class time where they can take exercise breaks. Options include stationary bicycles (which allow for studying while pedaling), Nerf ball activities, floor hockey, basketball shooting in the gym, and running around the gym. The program provides about 7 hours of physical activity per week. An external specialist visits regularly to guide students through the different activity stations. |
| 68 | Course 1km matin | Grades K-1 and 2 take a walk around the village every morning, during morning classes. If it rains, indoor activities are organized. |
| 69 | Ateliers de Taekwondo | Taekwondo workshops are offered during afternoon blocks to kindergarten students. Initially an extracurricular activity, it was later integrated into school hours to increase accessibility for younger children. The sessions are led by a teaching support staff member certified with a black belt in Taekwondo. |
| 70 | 20 minutes de mouvement | Each morning, teachers lead a 20-minute movement activity—such as running, walking, or organized play—to help students reach at least 60 minutes of daily physical activity. |
| 71 | Actif au quotidien | A training session for teachers was offered by the RSEQ, primarily targeting educators of the first cycle (Grades 1–2). The initiative aims to keep young students physically active even during class time. Its primary goal is to enable various stakeholders to understand and integrate a reference framework for psychomotor development in children aged 4 to 6 into their educational planning. The training supports: 1) Preschool teachers in integrating daily practices that promote the development of children’s psychomotor skills; 2) Daycare educators in incorporating routines that foster overall development, with a focus on increasing the intensity and variety of physical activities; 3) Physical education teachers in adopting a supportive and expert role within their schools regarding overall motor development.  Examples of activities carried out include:   - Hallways marked with colored tape to encourage movement such as hopping while navigating the school; - Encouraging students to work while standing or in various postures during class; - Organizing movement circuits within the classroom during frequent transitions (e.g., when retrieving materials).   <http://actifauquotidien.com> |
| 72 | Cours de piscine | All students attend swimming sessions 2 to 7 times annually during school hours, with older students participating more frequently. Kindergarten students receive certified “Swim to Survive” lessons. These sessions are part of a training program preparing students for a school-organized triathlon |
| 73 | Activité de ski de fond | A cross-country skiing activity is organized by the physical education teacher for all students three times a year during professional development days. |
| 74 | Olympiades de l’école | Every May, the school holds an Olympic-style event over one morning, where all students participate in activities such as a 100m run (or four laps around the park), hurdles, long jump, shot put, and skipping rope. This tradition has been celebrated annually for the past 10 years. |
| 75 | Portneuf en forme | The school board offers a program for remote areas in which specialist facilitators visit classrooms once a week to teach a physical exercise to both students and teachers. The class then repeats the exercise throughout the week. The program aims to promote physical activity and develop gross motor skills. Previously available for all grade levels, budget cuts have limited its availability to the first cycle (Grades 1–2). <https://services.qgdeportneuf.com/organismes/portneuf-en-forme> |
| 76 | Sortie Sportive | A joint activity day is organized with a neighboring school, featuring sports games, obstacle courses, and inflatable games to encourage student participation in a festive environment. The morning session is dedicated to first-cycle students (Grades 1–2). Parent volunteers assist throughout the day, and all parents are invited to return with their children between 3 p.m. and 5 p.m. to join in the races and games. |
| 77 | Course J’encourage! | An annual race takes place every October, where students run distances appropriate for their grade level. The event is supported by physical education teachers, other staff members, police officers, the municipality, and parent volunteers. |
| 78 | Course Communautaire à Obstacles | The school organizes an annual obstacle course event in partnership with a neighboring elementary school. Students from both schools participate, supported by parent volunteers, the city, police officers, firefighters, and an OBL that provides inflatable games for the day. |
| 79 | Club de Course | Students in grades 4 and 6 participate in a running club that begins training in January to prepare for a 42 km relay race at the end of the school year. Students form teams and each runs 1 km in relay format, with the entire school running the final kilometer together. <https://jeanxxiii.cssdgs.gouv.qc.ca/2017/02/01/le-club-de-course-de-lecole-jean-xxiii/> |
